# Separation and reconstruction of BCG and EEG signals during continuous EEG and fMRI recordings

**DOI:** 10.3389/fnins.2014.00163

**Published:** 2014-06-23

**Authors:** Hongjing Xia, Dan Ruan, Mark S. Cohen

**Affiliations:** ^1^Department of Bioengineering, University of California, Los AngelesLos Angeles, CA, USA; ^2^Department of Radiation Oncology, University of California, Los AngelesLos Angeles, CA, USA; ^3^Department of Psychiatry, Neurology, Radiology, Psychology, Biomedical Physics, California NanoSystems Institute, University of California, Los AngelesLos Angeles, CA, USA

**Keywords:** ballistocardiogram, simultaneous EEG-fMRI, artifacts, group sparsity, split Bregman, signal separation, segmentation

## Abstract

Despite considerable effort to remove it, the ballistocardiogram (BCG) remains a major artifact in electroencephalographic data (EEG) acquired inside magnetic resonance imaging (MRI) scanners, particularly in continuous (as opposed to event-related) recordings. In this study, we have developed a new Direct Recording Prior Encoding (DRPE) method to extract and separate the BCG and EEG components from contaminated signals, and have demonstrated its performance by comparing it quantitatively to the popular Optimal Basis Set (OBS) method. Our modified recording configuration allows us to obtain representative bases of the BCG- and EEG-only signals. Further, we have developed an optimization-based reconstruction approach to maximally incorporate prior knowledge of the BCG/EEG subspaces, and of the signal characteristics within them. Both OBS and DRPE methods were tested with experimental data, and compared quantitatively using cross-validation. In the challenging continuous EEG studies, DRPE outperforms the OBS method by nearly sevenfold in separating the continuous BCG and EEG signals.

## 1. Introduction

Concurrent acquisition of EEG and functional magnetic resonance imaging (fMRI) is an approach with great potential for studying different, yet connected aspects of cerebral activity, particularly bioelectric and hemodynamic attributes. With their different temporal and spatial resolutions, EEG and fMRI are understood to be linked functionally, and yet to hold complementary information regarding underlying brain activity. Simultaneous acquisition of both signals has proven its value in many applications, such as studies of spontaneous brain rhythms (Goldman et al., 2002), and the analysis of event-related brain responses (Mulert et al., 2004; Debener et al., 2005, 2006; Eichele et al., 2008; Moosmann et al., 2008; Sadeh et al., 2011; Diukova et al., 2012).

While artifacts in the simultaneously acquired MRI data now are relatively easy to manage (Huang-Hellinger et al., 1995; Allen et al., 2000; Goldman et al., 2000; Cohen et al., 2001; Cohen, 2002), artifacts appearing in the EEG data recorded inside the scanner presents a more challenging obstacle (Ullsperger and Debener, 2010; Mullinger et al., 2013). The most prominent magnetically induced artifact in EEG acquired inside the scanner is the ballistocardiogram (BCG) (Ullsperger and Debener, 2010; Yan et al., 2010; Mullinger et al., 2013). The BCG is especially difficult to suppress in protocols using continuous recordings, such as studies of the EEG rhythms. The BCG presents high temporal non-stationarity due to variation in cardiac cycles (Bonmassar et al., 2002; Debener et al., 2007), and its amplitude scales with magnetic field strength (Yan et al., 2010; Mullinger et al., 2013). This explains the considerable variation of success levels among studies, with more successful applications achieved at lower field strength.

Previously published methods to remove the BCG have approached the problem as one of blind source separation. At the time of this writing, the most widely used means of suppressing the BCG artifacts likely is the Optimal Basis Sets (OBS) method (Niazy et al., 2005), which uses principal component analysis (PCA) to identify components in the contaminated recordings, then adaptively removes the linear regression of the mean effect and a fixed number of components. This PCA-based algorithm therefore assumes orthogonality between the BCG and EEG subspaces, and that the selected principal components span the BCG subspace. Other widely used adaptive template approaches for BCG suppression such as Forbes and Fiume (2005) can be interpreted as weighted PCA to incorporate temporal model updates. Methods based on independent component analysis (ICA) (Srivastava et al., 2005; Ghaderi et al., 2010; Liu et al., 2012) also are used widely. All such blind source separation approaches, as reviewed in Grouiller et al. (2007) and Vanderperren et al. (2010), are limited to performing component extraction based on the contaminated data alone, agnostic of the structural difference between BCG and EEG.

Another approach to BCG suppression is to utilize reference signals for the artifact itself. Motion sensors (Bonmassar et al., 2002) and wire loops (Masterton et al., 2007) have been proposed to generate such reference signals. Recent developments (Mullinger et al., 2013; Xia et al., 2013; Chowdhury et al., 2014) utilize an insulating layer to directly acquire artifact signals from across the scalp. Although the measured artifact reference signals are not identical to the BCG (Mullinger et al., 2013), significant suppression can be achieved by a simple reference layer artifact subtraction (RLAS) (Chowdhury et al., 2014). However, RLAS requires purpose-built hardware and exploits no further denoising step besides a simple subtraction. We propose a method that has an experimental setup with no hardware modification and includes an additional denoising step using prior knowledge of EEG to further reduce residual BCG signals for continuous (non-ERP) experiments.

More specifically, we address the challenge of BCG artifact removal in spontaneous EEG-fMRI experiment from the perspective of subspace separation. Our method consists of two novel steps: (1) a basis analysis phase where representations of the BCG signal subspace and spontaneous (continuous) EEG signal subspace are characterized separately, and (2) a reconstruction phase where contaminated EEG data are decomposed into BCG and EEG components utilizing the learned bases, as well as structures of corresponding coefficients. For the basis analysis stage, we designed a new and simple recording configuration to obtain BCG-only signals directly inside the scanner, and clean EEG signals outside the scanner, alleviating the risk of model mismatches introduced by strong (and possibly impractical) assumptions about subspace relationships. In the reconstruction phase, we designed and implemented an optimization scheme that incorporated prior knowledge, more specifically the structures we discovered from studying pure BCG noise and clean EEG data individually, derived from our novel experimental setup. To assess the improvements we quantified the performance of the proposed method, and compared it with the OBS method, using both simulated and real contaminated data. In so doing, we demonstrated large improvements in BCG artifact removal.

## 2. Generative model for contaminated data

Though the exact cause of the BCG artifacts is not known completely, EEG and BCG signals are believed to originate from independent sources of different nature, as EEG arises from the brain, while BCG comes from physical movements of the head and blood. Basic electricity and magnetism dictates that the two signals should add linearly without interaction terms. Therefore, the contaminated measurements can be modeled as a superposition of BCG and EEG signals subject to noise contamination according to

(1) $$Y=X_{bcg}+X_{eeg}+\sigma$$

where *X*_*bcg*_, *X*_*eeg*_ and σ ∈ ℝ^*C* × *T*^ represent the BCG artifacts and the uncontaminated normal EEG brain signals acquired from our high-density system with noise σ. The dimension *C* = 256 is the number of channels in an EEG system, and *T* is the time points of the recordings. Moreover, the “independence” is in the sense of physics and physiology, rather than statistical. This generative model makes no presumption about the existence of their subspace relationships such as orthogonality or independence. This superposition model has been applied implicitly in many previous studies (Allen et al., 2000; Goldman et al., 2000; Niazy et al., 2005; Grouiller et al., 2007; Vanderperren et al., 2010).

## 3. Experimental setup

Three healthy adult volunteers, (2 males and 1 female, all right-handed, age between 24 and 26 years), gave informed consent for participation in this study according to the guidelines of the UCLA medical investigational review board. For our experiments, we used a 3T Siemens Tim Trio scanner (Siemens Medical Solutions, Erlangen, Germany). We acquired EEG data from both inside and outside the scanner using a GES300MR system (Electrical Geodesics, Inc., Eugene OR). This 256-channel apparatus made contact with the scalp via KCL-filled sponge contacts mounted in plastic pedestals with a contact-impedance of 20 kΩ or less. EEG data were sampled at 250 Hz and amplifier gains were kept constant. To focus on only BCG artifacts, no MRI scanning took place during the acquisition inside the scanner. The overall protocol was designed to record spontaneous brain activity with the focus on the variations of the alpha (8–13 Hz) EEG rhythm.

### 3.1. Experimental session I: acquisition of EEG-only data

Outside of the scanner room, we acquired EEG from one subject who lay comfortably inside an MR scanner simulator with earphones in place; sponge cushions were used to minimize head movements. The other two were studied in our electromagnetically-shielded EEG acquisition lab. All recordings were carried out in a darkened quiet environment, while the subjects lay supine on a carpet with a blanket, with a pillow made of viscoelastic foam placed under his head. They were asked to stay awake, with their eyes closed, during the whole acquisition. The simulator acquisition was designed to mimic closely the environment inside the scanner (subject posture and claustrophogenic aspects of the MR environment).

### 3.2. Experimental session II: acquisition of BCG-only and contaminated data

Inside the scanner, BCG-only and contaminated data were acquired at the same time from different channels in this session.

#### 3.2.1. Acquisition of BCG-only data

**Figure 1:**
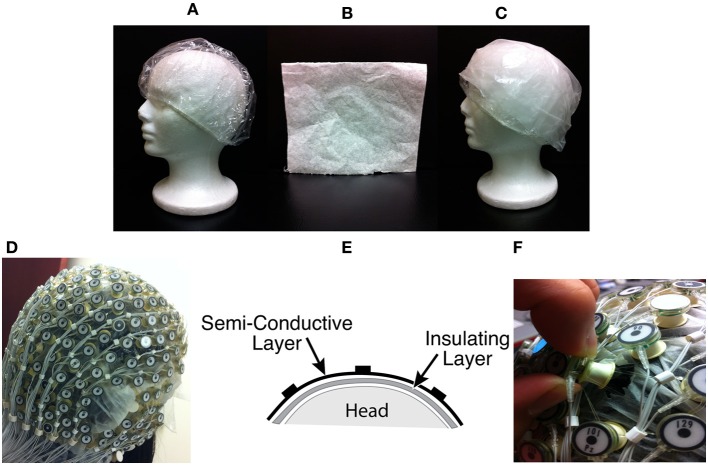
**(A)** Insulation layer: a shower cap **(B)** Semi-conducting layer: paper layer **(C)** A piece of thin paper dampened with saline placed on top of the insulation layer **(D)** A picture with all channels blocked **(E)** Sandwich diagram of construction **(F)** Unblocking one channel.

On the channels chosen to collect BCG-only signal, two layers of material were inserted between the scalp and the electrodes (Figures 1C,E).

1. Insulating Layer: To collect BCG-only artifacts, we first isolated a subset of electrodes from the scalp with a plastic insulating barrier to block brain signals from conduction, as shown in Figure 1A.
2. Semi-conducting Layer: In order to properly collect signals from insulated electrodes, a semi-conductive layer was then inserted between the insulating layer and electrodes. We chose a piece of thin paper dampened with saline (Figure 1B) as the semi-conductive layer, to provide proper impedance and to avoid any short circuit or alteration of BCG signals.

#### 3.2.2. Acquisition of contaminated EEG data

**Figure 2:**
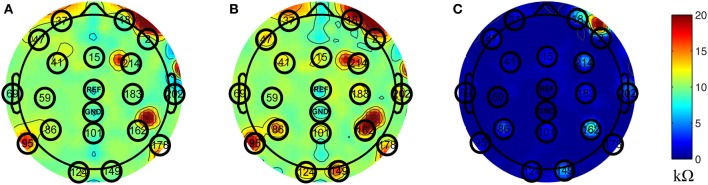
**Topographic maps of measured impedance**. The numbers shown on the topographic maps are channel numbers. The conventional channels along with the reference and ground channels are highlighted with black circles. The color indicates the measured impedance number in kΩ. **(A)** Measured impedance when all channels are blocked. **(B)** Measured impedance when reference and ground channels along with conventional 20 channels are unblocked. **(C)** The difference of impedance between **(A)** and **(B)**.

Inside the scanner, the unblocked channels recorded real EEG data corrupted by the BCG artifacts, simultaneously with the acquisition of BCG artifact-only signals from the blocked channels. We chose to block all channels globally, as shown in Figure 1D, and then unblock selected channels by removing the insulation and paper layers (see Figure 1F). As most experiments use standard low-density 10–20 systems to investigate the spontaneous brain rhythms, we chose to unblock 20 conventional channels, leaving 236 out of 256 channels blocked. This electrode-blocking pattern was chosen principally for its simplicity to demonstrate the feasibility of our new framework. One can determine which channels to block in advance and use the setup in Figure 1F to maximize the number of EEG channels that collect EEG signals. Figure 2A shows the measured impedance when all electrodes were blocked, including the reference and ground electrodes. Figure 2B is the measured impedance when the reference and ground channels are unblocked along with the 20 conventional channels. The impedance difference before and after unblocking is shown in Figure 2C. Note that the impedance of blocked and unblocked channels were all 20 kΩ or less, ensuring the quality of collected BCG-only signals.

## 4. Data preprocessing

Let *x* ∈ ℝ^*T*^ denote the collected data from one channel. We followed exactly the preprocessing procedure implemented in EEGLAB plug-in fMRI version 1.2 (Niazy et al., 2005). First, the slow drifts were removed with a high pass filter with a cutoff frequency at 1 Hz. Second, the filtered data *x* was segmented into *k* (*k* = number of heartbeats) segments, *x*_*i*_ ∈ ℝ^*m*^, for each detected heartbeat retrieved from the ECG channel. Each of these segments is an *m* × 1 column vector, where *m* is the number of time points within each heartbeat. Third, all segments were aligned in a matrix $\tilde{X}$ = [*x*_1_ | *x*_2_ | … | *x*_*k*_] ∈ ℝ^*m* × *k*^. Finally, the mean effect *x* = $\frac{1}{k}\sum_{i\;=\;1}^{k}x_{i}$ ∈ ℝ^*m*^ was calculated for all segments and removed from the data matrix before a PCA was applied to the residual artifacts, **X**. The same procedure was applied to all collected data including BCG-only, EEG-only and contaminated signals. While alignment to the heartbeats facilitates learning of the BCG bases by reducing the data complexity caused by the non-stationary heartbeats, it has no obvious advantage for the EEG data.

Unlike the OBS method, where the mean effect along with the first several PCs were fitted to, and subtracted from, each segment of contaminated data, **Ỹ**, to generate the estimated EEG signals, our method operated by separating the demeaned BCG, **X**_*b*_, and EEG, **X**_*e*_, matrices from the demeaned contaminated data, **Y**. The mean effect derived from **Ỹ**, was added back to the recovered BCG matrix under the assumption that the EEG signals are close to zero-mean, as EEG segments should be relatively uncorrelated with the heartbeats. The same assumption is made in the OBS method (Niazy et al., 2005).

We use **X**[:, *j*] to denote the *j*th column vector, and **X**[*i*, :] for the *i*th row vector of matrix **X**. Subscripts are used to indicate the type of signals. As prior information for the BCG and EEG signals, the pure BCG from one channel (B) in session II is denoted as **X**_*b*_*prior*_ ∈ ℝ^*m* × *k*^_1_ and the EEG in session I, from another channel (A or B), is denoted as **X**_*e*_*prior*_ ∈ ℝ^*m* × *k*^_2_. To minimize spatial variations of the BCG artifacts we chose the BCG prior data from a channel adjacent to the contaminated data as well as the BCG data used in the following simulation. This adjacent channel was placed to avoid major surface vessels.

## 5. Stage I: basis construction

Unlike the OBS method, where basis vectors are retrieved from contaminated data, our direct-recording prior encoding (DRPE) approach generates them from the experimentally acquired BCG-only and EEG-only signals. We expect direct characterization of the BCG and EEG subspaces to be advantageous, in that they remain more faithful to each signal type. We use principal component analysis (PCA) in this pilot investigation. The prior data matrices for BCG (**X**_*b*_*prior*_) and EEG (**X**_*e*_*prior*_) correspond to the following decomposition:

(2) $$\begin{array}{l} X_{b\_prior}=\;B_{b\_prior}C_{b\_prior}\\ X_{e\_prior}=\;B_{e\_prior}C_{e\_prior} \end{array}$$

where **C**_*b*_*prior*_ and **C**_*e*_*prior*_ are coefficient matrices, and **B**_*b*_*prior*_ and **B**_*e*_*prior*_ are basis matrices with orthonormal columns. The resulting PCA basis, **B**_*e*_*prior*_, expands a subspace that best incorporates all possible phases presented in this training session. One may envision the basis set as a set of typical temporal signatures, and the specific phase in each segment is captured by the variation in the weighting coefficients.

### 5.1. Justification of BCG prior basis vectors

**Figure 3:**
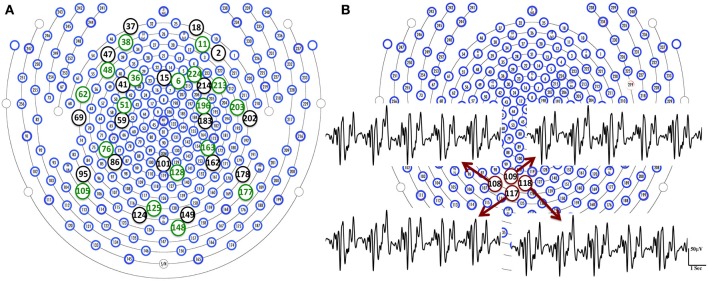
**(A)** We are able to find a channel (green circle) that gives the smallest relative error in the neighborhood of each target channel (black circle). **(B)** BCG temporal traces from four neighboring channels are displayed with highlighted channel location in red circles.

BCG artifacts can be caused by the magnetic flux changes from either the magnetic field or wire loop movement from either local electrodes movement or more global head rotation (Yan et al., 2010). Surface blood flow is an example of the former, while the latter include respiration-induced movement of electrodes, and pulsation of blood vessels. In the our experimental setup, using the 256-channel collection net, we expect surface blood flow velocities, and the electrode movements, to be locally consistent, given the close placement of the neighboring electrodes in the dense net. With the BCG from the fully blocked net, the relative errors (*RE*) between BCG signal from each target channel (here we use conventional 20 channels for illustration-purposes), and those from the remaining channels, are calculated. We show in Figure 3A that each target channel has a corresponding neighboring channel that gives the smallest relative error. Figure 3B reveals the similarities of BCG traces among four neighboring channels. It is therefore safe to assume that for any channel under examination, there exists a neighboring blocked channel whose BCG reading closely resembles the BCG artifact from the unblocked channel. This is ensured further by creating blocking patterns that provide a sufficient number of adjacent ground-truth BCG signals as candidates for this purpose.

### 5.2. Justification of EEG prior basis vectors

Although the EEG measurements recorded inside and outside of the MR scanner may not be exactly equivalent (Sammer et al., 2005; Debener et al., 2006), potentially being affected by the posture, and by the magnetic and claustrophobogenic MR scanner environment, it still is reasonable to assume that the brain EEG generates consistent recordings both inside and outside the scanner, produces similar EEG characteristics, such as the dimensionality of normal brain EEG data and global power spectrum. For ERP-type EEG signals, a different prior should be considered, as the timing of triggering events is available. For continuous EEG signals, we opt for a weak prior in terms of a consistency requirement. In continuous EEG, one does not have access to strong structural alignment references such as trigger timing in ERP, and has to rely on weaker consistency type priors for signal modeling. Here we assume the EEG signal representation space is approximately consistent, and extrapolate from the bases learned from outside the scanner to estimate the EEG-only signal inside the scanner. The difference in the signal, *per se*, and the temporal non-stationarity, is characterized by the variation in the weighting coefficient with respect to the basis. In other words, we utilize the same set of basis functions for EEG signal acquired outside and inside scanner, but with different composition weightings.

On the experimental level, we have tested our signal separation power (see section 8.3) with bases learned from different subjects under different acquisition environments (with or without a mock scanner), keeping only the posture of subjects the same. Furthermore, on the theoretical level, we use the basis function, rather than the data themselves, from EEG-only collected data outside of the scanner, to facilitate inside scanner reconstruction and analysis: this requires only a rough consistency of signal space, rather than strict equivalence.

## 6. Stage II: separation and reconstruction

Based on the assumption that the characteristics of continuous EEG signals generated inside and outside the scanner are reasonably consistent for the same subject, and that the BCG signals acquired from our insulated channels are similar to the BCG components in the unblocked channels, we propose to reconstruct the BCG/EEG components from the contaminated data by estimating the coefficients for the bases from BCG (inside the scanner) of a neighboring channel and EEG (outside the scanner) from the same subjects.

### 6.1. Regularizations for reconstruction

#### 6.1.1. BCG

**Figure 4:**
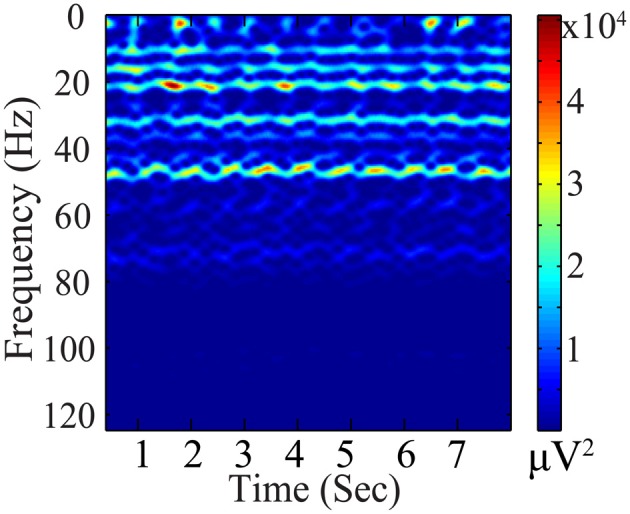
**Spectrogram (μ ***V*****^2^**) of BCG artifacts (from one channel)**.

Significant temporal variations exist in the BCG artifacts, as illustrated in Figure 4. Based on the premise that the reconstructed BCG should be similar to the concurrently acquired BCG-only signals from the blocked electrodes (**X**_*b*_*prior*_), we chose an ℓ_2_ penalty as the first term in the minimization objective in Equation (3).

#### 6.1.2. EEG

**Figure 5:**
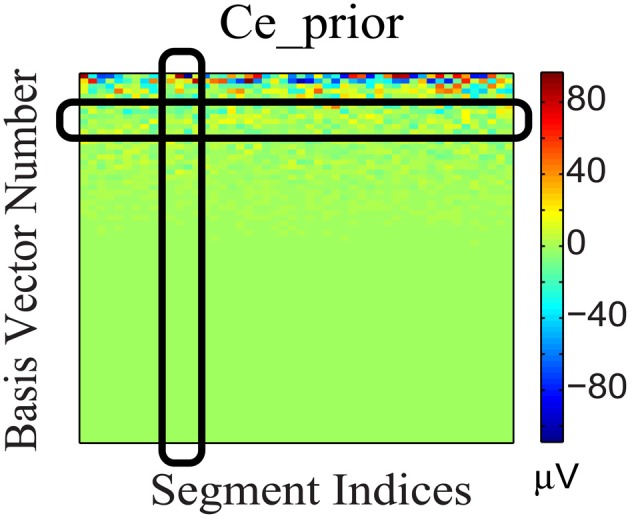
**Coefficient matrix (**C*_*e*__***_***prior***_) of the EEG prior data**. The black column and row highlights show that **C**_*e*_*prior*_ is sparse in basis vector representation (column) and dense across segments (row).

To incorporate the likely temporal non-stationarity of the continuous EEG signal (as shown in Figure 5), we impose a flexible prior based on a general low-dimensionality argument. We expect the EEG signals to span only a small number of bases. This not only is consistent from the perspective of dipole model, but it can be validated further by analyzing the non-white continuous EEG data acquired from outside the scanner, which have low intrinsic dimensionality. Furthermore, the variation of coefficients across different segments (rows) may be due to phase changes, which we attempt to preserve: Segments with similar phases would correspond to similar distribution patterns of significant coefficients, as the bases are the same for all segments. Therefore, (1) each EEG segment should be represented as the superposition of a few bases (corresponding to sparsity along column direction of the coefficient matrix); and (2) the distribution of the significant coefficient values is dense along the temporal (segment-indexing) direction, represented as dense rows, because the same bases are involved in the generation of phase shifts. These considerations gave rise to a structural regularization of the group-sparsity type (Deng et al., 2011) whose columns are sparse, and whose rows are dense. Mathematically, this can be achieved by imposing a weighted group sparsity penalty with ℓ_2,1_ (or ℓ_*w*,2,1_) norm, ${\left\|C_{e}\right\|}_{w,2,1}\overset{def}{=}\;\sum_{i\;=\;1}^{m}\;w_{i}\;\|C_{e}\;[\;i,\;:\;]{\|}_{2}$, on the reconstructed EEG coefficient **C**_*e*_, where *i* ∈ {1, …, *m*} is an index set indicating the *i*th group (row), and *m* is the number of rows in **C**_*e*_. The weights *w*_*i*_ ≥ 0 are associated with each group. Given these considerations, we expect the group-sparsity regularization to help steer the coefficient estimates toward a more favorable reconstruction.

### 6.2. Objective function

Let **Y** denote the contaminated data from a target channel, A, with unknown BCG component, **X**_*b*_, and unknown EEG component, **X**_*e*_. A neighboring channel, B, is blocked, and its BCG-only signals are recorded concurrently to provide prior BCG basis **B**_*b*_*prior*_, and coefficients **C**_*b*_*prior*_. The prior EEG basis, **B**_*e*_*prior*_, (from either channel A or B) comes from the recordings made in experimental session I. These considerations yield an overall reconstruction model:

(3) $$\begin{array}{l} \underset{C_{b},C_{e}}{\min}\lambda \;\left\|\;C_{b\_prior}-C_{b}\right\|{\;}_{F}^{2}+\;\left\|\;C_{e}\;\right\|{\;}_{w,2,1}\\ \text{s.t.}Y=B_{b\_prior}C_{b}\;+\;B_{e\_prior}C_{e} \end{array}$$

where λ is a parameter to balance the BCG and EEG prior contributions. We apply the alternative direction method of multipliers (ADMM) to solve the augmented Lagrangian problem of our reconstruction model (see the supplementary material). After obtaining the estimated coefficients (**C**_*b*_ and **C**_*e*_), we proceed to recover the BCG and EEG of target channel A by multiplying those with the basis vectors from the training data,

(4) $$\begin{array}{l} {\hat{X}}_{b}=B_{b\_prior}C_{b},\\ {\hat{X}}_{e}=B_{e\_prior}C_{e}. \end{array}$$

## 7. Results from synthesized contaminated data

**Figure 6:**
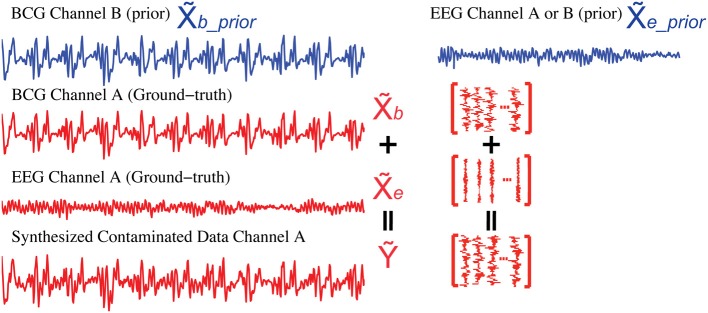
**Synthesis of contaminated data**. We use the BCG data from channel B (blue) as the prior BCG data denoted as $\tilde{X}$_*b*___*prior*_ after alignment. The EEG data from channel A or B (blue) can be used as the prior EEG data $\tilde{X}$_*e*_*prior*_. Simulated contaminated data (**Ỹ**) is summed from the BCG ($\tilde{X}$_*b*_) and EEG ($\tilde{X}$_*e*_) data (red) both from channel A.

To evaluate different artifact removal approaches quantitatively, and to provide parameter selection guidance when real contaminated data is used, we simulated contaminated EEG data from known BCG-only and EEG-only recordings according to the generative model introduced in section 2, allowing direct comparison between reconstructed and ground-truth component signals. First, we selected *k*_1_ segments of EEG-only signals as ground-truth EEG (the red EEG recordings in Figure 6), denoted as $\tilde{X}$_*e*_ ∈ ℝ^*m* × *k*^_1_, which were acquired from one unblocked channel A outside the scanner in session I. Then, the ground-truth BCG-only signals, denoted as $\tilde{X}$_*b*_ ∈ ℝ^*m* × *k*^_1_, were chosen from the acquisition of channel A from inside the scanner in session II. Finally, the contaminated data, denoted as **Ỹ** ∈ ℝ^*m* × *k*^_1_, were synthesized according to the generative model **Ỹ** = $\tilde{X}$_*b*_ + $\tilde{X}$_*e*_. Figure 6 illustrates this process. Notice that the EEG-only signals, $\tilde{X}$_*e*_*prior*_, were from either channel A or B (a neighbor of channel A) recorded at a time different than that used for EEG data, $\tilde{X}$_*e*_, in simulating the contaminated data **Ỹ**; BCG prior data, $\tilde{X}$_*b*_*prior*_, were collected at the same time as $\tilde{X}$_*e*_ and $\tilde{X}$_*b*_, but from channel B.

### 7.1. Assessment of validity of orthogonal assumption between subspaces

**Figure 7:**
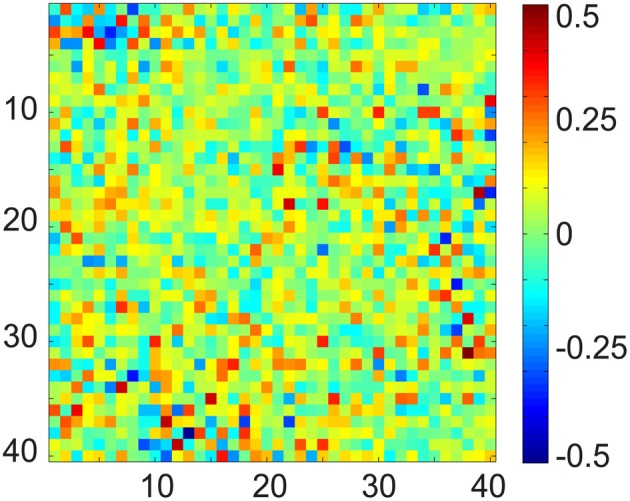
**Matrix product of the normalized BCG and EEG basis vectors using up to 40 PCs**. In fully orthogonal subspaces the expected value of al elements would be zero, whereas here the values are large.

To demonstrate the limitation of OBS, and to motivate our effort to develop a more realistic and data-driven approach in DRPE, we first checked the relationship of the BCG and EEG subspaces. The OBS method generates bases from contaminated data, and its reconstruction follows the assumption that the first several sequential PCs approximate the subspace of the BCG. The residual of projecting onto the span of segment-wise mean, and the PCs, yields the EEG component. This rationale assumes implicitly that the BCG and EEG subspaces are approximately orthogonal. Without ground-truth BCG- and EEG-only signals, there is no good way to test the feasibility of the assumption. Our experimental data from section 3 provides observations of these BCG- and EEG-only signals, and offers an opportunity to examine the validity of the assumptions of OBS, and to explore further methodological improvements. In Figure 7 we show the multiplication (**B**^⊺^_*b*_*prior*_**B**_*e*_*prior*_) result from up to 40 of the BCG and EEG basis vectors (PCs) from prior BCG and EEG data, $\tilde{X}$_*b*_*prior*_ and $\tilde{X}$_*e*_*prior*_. The [*i*, *j*]^*th*^ element value of this matrix is the inner product of the *i*^*th*^ basis of BCG and the *j*^*th*^ basis of EEG. Complete orthogonality of the EEG and BCG subspaces would correspond to a matrix containing only zero elements. The fact that the matrix of Figure 7 contains many significant values of PC interaction, especially in its upper left corner, indicates that the assumption of orthogonality of the BCG and EEG subspaces is invalid, and necessitates the development of denoising methods beyond OBS.

### 7.2. Performance evaluation of reconstruction

**Figure 8:**
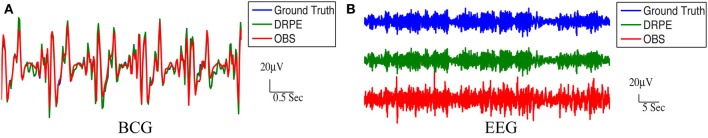
**Comparison of the reconstruction results from DRPE and OBS**. Ground truth, DRPE and OBS results are shown in each panel. The DRPE method yields only 6.685% and 16.45% relative errors for the BCG and EEG reconstruction, while the OBS generates 47.50% and 117.5% relative errors for BCG and EEG. The reconstructed BCG **(A)** from DRPE almost overlaps with the Ground truth BCG. The large spikes in reconstructed EEG **(B)** from OBS are due to the residual BCG signals.

**Figure 9:**
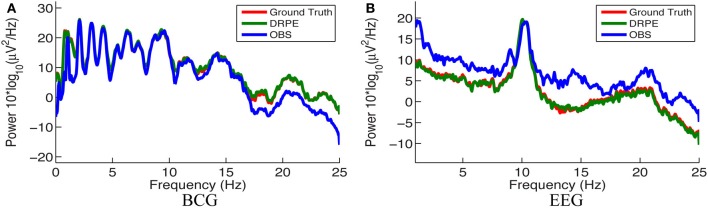
**Comparison of the frequency spectra of reconstructed BCG (A) and EEG (B) signals from the DRPE and the OBS methods as well as their corresponding ground-truth data**.

We quantified the signal separation performance of DRPE and OBS method, in terms of relative error *RE*, defined as *RE* = ‖ $\hat{X}$ − **X**_*truth*_ ‖ _*F*_/ ‖**X**_*truth*_ ‖ _*F*_. Recovered BCG and EEG components, and their corresponding ground-truth, are represented with $\hat{X}$ ∈ ℝ^*m* × *k*_1_^ and **X**_*truth*_ ∈ ℝ^*m* × *k*_1_^, respectively. The results from the DRPE method were derived with parameters (λ = 10^−2.4^, β_1_ = 10^−6.4^ and β_2_ = 10^4.2^), and all of the results of the OBS method were obtained from the OBS implementation in the EEGLAB plug-in fMRI version 1.2 (Niazy et al., 2005) with the number of PCs (including the mean vector) set to 3 (*N*_*pc*_ = 3). The contaminated data were simulated from 13.6 min BCG-only and EEG-only data both from channel A. We learned the prior BCG basis vectors from BCG-only data concurrently from a neighboring channel, B, and learned the prior EEG basis vectors from non-concurrent EEG-only data from channel B from a different 8.9 min time segment. To reduce the computational burden, we down-sampled all data from 250 to 50 Hz (with an anti-aliasing filter as explained in section 4.) After aligning the recordings to the detected heartbeats, the resulting data from the 13.6 and 8.9 min, recordings were re-formed as matrices of size 73 × 848 and 73 × 556, respectively. Figure 8 shows a typical portion of the reconstructed results from the two methods, alongside the ground-truth data. The differences between the reconstructed signals and the ground truth ones are in the supplementary material. Figure 9 shows the corresponding frequency spectra. It is clear that the BCG and EEG components are much better separated and preserved by the DRPE method; the relative errors for EEG components are reduced by approximately sevenfold. Figure 8 shows as well that the DRPE successfully recovers the qualitative temporal behavior of the EEG signals much better than does OBS.

We further employed a standard threefold cross-validation (Friedman et al., 2001) to quantify the overfitting and the consistency of our DRPE method, and compared the results to that of OBS (with *N*_*pc*_ = 3). Letting *Y*^(1)^ denote a 73 × 251 (4.5 min) matrix containing a randomly selected subset of column vectors from synthesized contaminated matrix **Y** (13.6 min), and letting **Y**^(1)^ be a 73 × 607 matrix (9.1 min) containing the complementary set of data vectors used for training parameters we then applied DRPE and OBS to recover BCG and EEG components from the **Y**^(1)^. The parameters (λ, β_1_, and β_2_) of the DRPE were tuned for the best recovery of the EEG components. Once the optimal parameters were determined for the training dataset **Y**^(1)^, they were used in recovering the BCG and EEG signals from the validation dataset **Y**^(1)^. The process was then repeated using three non-intersecting subsets of the data to calculate reconstruction errors of the training and validation for each subset. The relative errors of the cross-validation process are listed in Table 1. Selected parameters in the table are relatively consistent, and result in similarly good reconstruction results for all the training sets, with only slightly worse results for the validation sets. This strongly suggests that the DRPE method is stable, with nearly negligible overfitting.

**Table 1:** **Cross validation results from three groups: relative errors (RE) in percentage**.

**RE(%)**	**DRPE**							**OBS**			
	**BCG segments**		**EEG segments**		**Parameters**			**BCG segments**		**EEG segments**	
	**T**	**V**	**T**	**V**	**log_**10**_ λ**	**log_**10**_ β_**1**_**	**log_**10**_ β_**2**_**	**T**	**V**	**T**	**V**
Group 1	6.66	6.90	16.53	17.18	−2.2	−6.2	−3.6	52.31	61.50	130.5	153.5
Group 2	6.78	6.96	16.69	16.98	−2.2	−6.2	−4.2	52.47	61.54	129.9	150.6
Group 3	6.70	6.88	16.42	16.90	−2.2	−6.2	−4.2	51.97	61.02	128.0	150.5

Each group has a different segment as validation set with the remaining two segments as training set.

T: Training Session. V: Validation Session.

β_1_ and β_2_: penalty parameters for the corresponding augmented Lagrangian problem (see supplementary material).

## 8. Results from real contaminated data

One of the most robust effects on the EEG results from signal comparisons of eyes-closed (EC) and eyes-open (EO) states at rest, which results in large alpha band increases in the EC condition (Berger, 1929). Without access to ground-truth EEG-only signals acquired inside a scanner, we demonstrated the feasibility and advantage of our DRPE method on real contaminated data in an EC/EO paradigm using parameters selected from the same simulation process above, whose contaminated data was composed of EEG-only signals acquired outside the scanner, and BCG-only signals acquired *inside* the scanner.

### 8.1. Experimental setup

We acquired new data for both experiment session I and II when the subject was cued verbally to open and close his or her eyes every 30 s, for a total time of 15 min for each session.

### 8.2. Statistical analysis

We followed the procedure of Chen et al. (2008) to quantify the EC/EO effects. Each 30 s EEG sample, omitting 2 s before and after each EC/EO event onset, was analyzed in 3 s epochs, resulting in 112 epochs for each EC/EO state, from a total 14 min recordings. The absolute EEG band power (μ *V*^2^) in the alpha band from each epoch of EC/EO state was calculated using the Fast Fourier Transform. As the alpha band power values failed a normality test, Wilcoxon test for non-parametric comparison of ranks was performed, with *p* < 0.05 accepted as significant, to assess the hypothesis that EC and EO states have similar population mean rank based on alpha band power (Chen et al., 2008).

### 8.3. Effects of states from reconstructed signals

**Figure 10:**
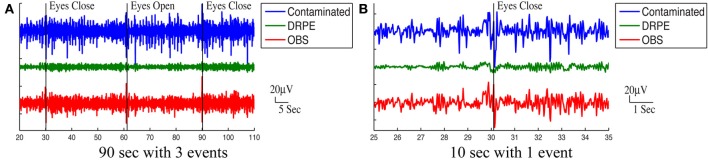
**Roughly 90 s and 10 s ranges of reconstructed EEG signals (in μ ***V***) were shown here with events (eyes open and eyes close)**. In **(A,B)**, top panel: reconstructed EEG signal from the DRPE method. Bottom panel: reconstructed EEG signals from the OBS method.

**Figure 11:**
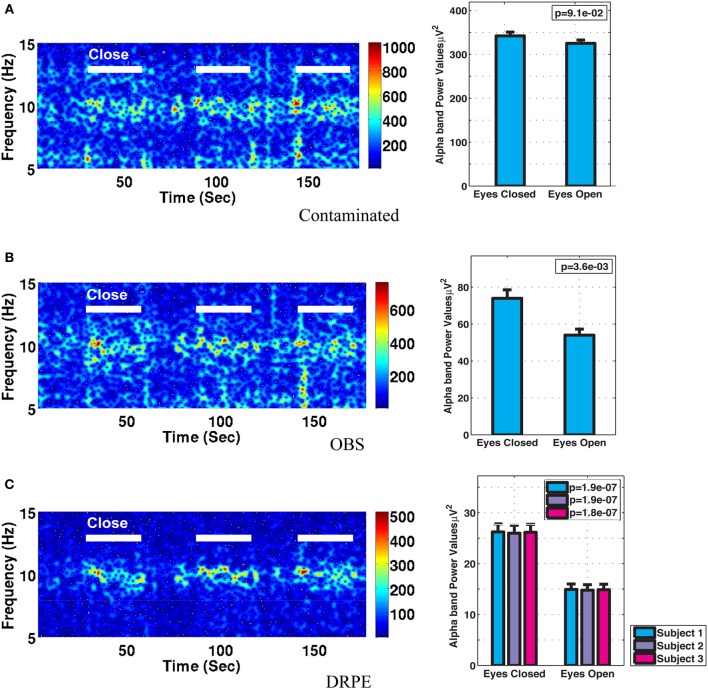
**Comparison of performance in differentiating the eyes open (EC) and eyes closed (EO) states**. **(A)** Based directly on contaminated EEG recording, **(B)** recovered EEG signals with the OBS method, and **(C)** the proposed DRPE method. The left panel depicts the reconstructed spectrograms. The right panel displays the Wilcoxon rank test results of alpha band power comparisons between the EO and EC states; standard errors are indicated. No significant change in alpha power was detected in the contaminated signal, while both the DRPE and OBS methods display the expected decreases from EC to EO conditions. In **(C)**, the EC and EO results are compared when the EEG basis was derived from subject 1 (blue) or from subject 2 and 3 (purple and red). The reconstruction results were virtually identical when the EEG bases were derived from the original subject, or from the other two participants, recorded in different environments.

Figure 10 shows the reconstructed EEG signals from real contaminated data of channel 124 with prior BCG and EEG data sampled from neighboring channel 137. Parameters used in reconstruction were selected from the simulation process, but using the new BCG- and EEG-only data from experiment sessions I and II. The differences in alpha energy between the EC to EO states can be identified clearly at around 30, 60, 90 s… as the subject closed and opened his eyes. With both the BCG and EEG prior data from the neighboring channel, the reconstructed EEG signals of channel 124 from the DRPE method have shown more prominent distinctions than OBS, between the EC and EO states. When the contaminated data were used (EC: mean 342.0, median 328.0 μ *V*^2^, EO: mean 325.1, median 310.2 μ *V*^2^; *p* = 0.09 > 0.05), our test indicated no significant reduction in the alpha band power in the EO, as shown in Figure 11A. By contrast, statistically significant differences (Figure 11B) between the EC and EO states were present in the magnitude of the alpha band power in the recovered EEG signals from OBS (EC: mean 73.95, median 66.87 μ *V*^2^, EO: mean 54.02, median 45.59 μ *V*^2^; *p* = 0.0036 < 0.05), agreeing with the results in the original OBS paper (Niazy et al., 2005). In addition, the EEG signals estimated from DRPE also reveal statistically significant difference (Figure 11C) between the EC and EO states (EC: mean 26.23, median 26.07 μ *V*^2^, EO: mean 14.93, median 11.38 μ *V*^2^; *p* = 1.9 × 10^−7^ < 0.05). To test the robustness of the DRPE method in terms of basis characterization, we have (1) applied EEG prior acquired from one subject to the BCG denoising for another subject, (2) used EEG prior acquired in a normal EEG room without the mock scanner. For the DRPE method, the EEG prior signals from two other subjects were also employed, both of which were acquired when subjects lying in a normal EEG room without mock scanner. A spectrogram of the EEG signals, and the corresponding Wilcoxon test results, are displayed in Figure 11.

## 9. Discussion and conclusions

Removing BCG artifacts from contaminated EEG data is a major bottleneck for the successful integration of simultaneously recorded data. First, the BCG component (magnitude >200 μ V) often dominates the EEG component (10–100 μ V) in the contaminated signal by an order of magnitude (Yan et al., 2010). Second, the BCG artifacts show considerable temporal variation as shown in Figure 5 (Huang-Hellinger et al., 1995; Bonmassar et al., 2002; Debener et al., 2007). Third, the BCG and EEG subspaces have a complex geometric relationship with non-trivial overlap that violates the assumption of simple mutual-orthogonality (c.f. section 7.1) making common approaches, such as PCA, both inappropriate and ineffective. Finally, unless ground-truth BCG data are accessible, overfitting and relative error of recovered signals cannot be quantified directly (Niazy et al., 2005; Grouiller et al., 2007; Vanderperren et al., 2010).

When both BCG and EEG signals exist, accurate representations of the subspaces are necessary to decompose contaminated signals. Powerful, and widely used, the optimal basis sets (OBS) method of Niazy derived the subspace representations solely from contaminated signals, but it relies on questionable presumptions (e.g., orthogonality) about the BCG and EEG subspaces. By contrast, our procedure enables separate access to BCG and EEG subspaces, providing more accurate basis vectors for the purpose of reconstruction. Our DRPE approach is based on the assumption that, in the case of continuous spontaneous EEG experiments, the EEG basis learned from outside the scanner is a sufficient representation of the EEG component measured inside the scanner. This allows us to facilitate the separation of BCG and EEG using prior knowledge of the coefficient structures of the EEG, and neighboring BCG-only, signals. Moreover, we have demonstrated (Figure 11) that the EEG bases learned from different subjects and acquisition environment are sufficiently consistent for effective denoising. We recognize that the challenges of ERP signals and continuous EEG signals differ: with knowledge of triggering event timing information, we are designing a different type of prior and objective function to take advantage of the problem structures. One possible limitation of the present work lies in the additive generative model of the contaminated data in Equation (1). While we cannot verify this directly, there is little reason to believe that strong interactions couple EEG and BCG in the biologically recorded signals. The effectiveness of recovering EEG signals is demonstrated here by its application to real contaminated data from an eyes open/eyes closed study with denoising parameters tuned from the simulation study.

In addition to providing more representative basis vectors, the DRPE method yields a novel means to introduce structures on the coefficient sets. In particular, penalty functions are designed to regularize the temporal pattern of the BCG by the ℓ_2_-norm, and the group characteristics of EEG coefficients by ℓ_2,1_-norm. The feasibility of enforcing ℓ_2_ and ℓ_2,1_-regularizations is demonstrated qualitatively and quantitatively by our studies on both simulated and real contaminated data. In our evaluation of the contaminated data simulated from BCG-only and EEG-only signals, the relative errors of the reconstructed BCG and EEG data are as low as 6.685 and 16.45%, compared to 47.50 and 117.5% from the benchmark OBS method. Notably, both the BCG and EEG priors are acquired from an adjacent channel, while the EEG priors were obtained from outside the scanner. This demonstrates that the prior data is relatively insensitive to small spatial location changes across different experiment sessions. It suggests also the potential of extending this result to the whole head, by creating blocking patterns that provide multiple BCG channels. Our recording configuration is compatible with various blocking patterns, and we are in the process of evaluating the merits of different options.

Moreover, the DRPE method can be integrated with other approaches that generate BCG reference signals—some potential candidates are the more recently developed reference layer artifact subtraction (RLAS) method (Chowdhury et al., 2014) and others (Bonmassar et al., 2002; Masterton et al., 2007) which have provided alternative means to record BCG reference signals. Admittedly, there exists some discrepancy between each of these reference signals and the “ground-truth” BCG signals, as a result of either insulation or sensing process. While these signal differences become the limiting error term (Ullsperger and Debener, 2010) when used simply for linear subtraction, DRPE utilizes the reference signals as statistical priors, and flexibly compensates this discrepancy with the incorporation of priors built on continuous EEG from outside the scanner. The RLAS method potentially will alleviate the needs to find consistent neighboring channels that provide BCG reference for the DRPE method. In principal, Hall effects occurring in the MR imaging field might distort the scalp topography of the EEG signals. It is difficult to estimate the magnitude of this contaminant, which is common to OBS and DRPE.

Our recording configuration enables quantitative comparison of various artifact removal techniques. We used the Frobenius-norm, which resembles the root mean square error, to quantify the difference between reconstructed signals and their corresponding source signals. The relative errors facilitate the comparison of the results among different signal type. Here, we used this approach, and K-fold cross-validation, to quantify and compare the DRPE and OBS methods.

Although PCA is used in this paper to generate the basis matrix for each of the EEG and BCG subspace representations, other representations, such as ICA, can be substituted without affecting the integrity and compatibility of the recording configuration for subspace-specific data collection and the reconstruction process, though different basis representation may give rise to different coefficient behavior, and the objective function of Equation (3) would need to be designed accordingly. We expect different choices of basis representation to affect the reconstruction performance, and it is our next step to optimize over such representations.

Preliminary tests have demonstrated the feasibility and efficacy of the proposed approach. There are a few practical issues for the clinical applications of this new method. First, the computational demand is high compared to the OBS method, and subject-specific parameter optimizations may be necessary. The alternating direction method of multipliers (ADMM) we applied in solving the objective function (see the supplementary material) takes 1–2 s to evaluate each set of parameters. We expect, however, that this problem will yield readily to computational optimizations. Second, the additional time needed to acquire clean EEG data could impose some burdens in clinical or research studies. Improvement of workflow might be able to minimize this impact.

### Conflict of interest statement

The authors declare that the research was conducted in the absence of any commercial or financial relationships that could be construed as a potential conflict of interest.
