# Supplementary material for: Separation and reconstruction of BCG and EEG signals during continuous EEG and fMRI recordings
Source: Front Neurosci. 2014 Jun 23;8:163. doi: 10.3389/fnins.2014.00163 (PMC4067090; doi:10.3389/fnins.2014.00163)
Supplement: Supplementary file 1 [file DataSheet1.PDF]

Frontiers in Brain Imaging Methods

Research Article
19 June 2014

Supplementary Material: Separation and Reconstruction of BCG and EEG Signals during Continuous EEG and fMRI Recordings

Hongjing Xia [1,*], **Dan Ruan** [1,2] **and Mark S. Cohen** [1,3]

[1] *Department of Bioengineering, University of California, Los Angeles, CA, USA*
[2] *Department of Radiation Oncology, University of California, Los Angeles, CA, USA*
[3] *Department of Psychiatry and Psychology, University of California, Los Angeles, CA, USA*

Correspondence*:
Hongjing Xia
Department of Bioengineering, B8-169 Semel Neuropsychiatric Institute, University of California, Los Angles, 760 Westwood Plaza, Los Angeles, CA 90095-8353, USA, xiahongjing@ucla.edu

1 SUPPLEMENTARY DERIVATIONS

We use a split Bregman method **Cai et al.** (2009); **Goldstein and Osher** (2009) (also known as augmented Lagrangian method) to solve this problem. In particular, by introducing an auxiliary variable $\mathbf{Z}$, the primary problem equation (1)

$$\begin{aligned} &\min_{\mathbf{C}_b,\mathbf{C}_e} \lambda \left\|\mathbf{C}_{b\_prior} - \mathbf{C}_b\right\|_F^2 + \|\mathbf{C}_e\|_{w,2,1} \\ &\text{s.t.}\mathbf{Y} = \mathbf{B}_{b\_prior}\mathbf{C}_b + \mathbf{B}_{e\_prior}\mathbf{C}_e \end{aligned} \tag{1}$$

is equivalent to:

$$\begin{aligned} &\min_{\mathbf{C}_b,\mathbf{Z}} \|\mathbf{Z}\|_{w,1,2} + \lambda \left\|\mathbf{C}_{b\_prior} - \mathbf{C}_b\right\|_F^2 \\ &\text{s.t. } \mathbf{Y} = \mathbf{B}_{b\_prior}\mathbf{C}_b + \mathbf{B}_{e\_prior}\mathbf{C}_e \\ &\quad \mathbf{Z} = \mathbf{C}_e \end{aligned} \tag{2}$$

The corresponding augmented Lagrangian problem is of the form:

$$\begin{aligned} &E(\Lambda_1, \Lambda_2, \mathbf{Z}, \mathbf{C}_b, \mathbf{C}_e) = \\ &\|\mathbf{Z}\|_{w,1,2} + \left\|\mathbf{C}_{b\_prior} - \mathbf{C}_b\right\|_F^2 - < \Lambda_1, \mathbf{Z} - \mathbf{C}_e > \\ &+ \frac{\beta_1}{2} \|\mathbf{Z} - \mathbf{C}_e\|_F^2 - < \Lambda_2, \mathbf{B}_{b\_prior}\mathbf{C}_b - \mathbf{B}_{e\_prior}\mathbf{C}_e - \mathbf{Y} > \\ &+ \frac{\beta_2}{2} \left\|\mathbf{B}_{b\_prior}\mathbf{C}_b - \mathbf{B}_{e\_prior}\mathbf{C}_e - \mathbf{Y}\right\|_F^2, \end{aligned} \tag{3}$$

where $< .,. >$ is the inner product, Λ_1 and Λ_2 are Lagrange multipliers, while $\beta_1, \beta_2 > 0$ are penalty parameters. We then apply the classic alternating direction method (ADM) to solve the augmented Lagrangian problem with respect to $\mathbf{Z}$, $\mathbf{C}_b$ and $\mathbf{C}_e$ alternately.

In short, we have derived an ADM iteration scheme as follows:

Algorithm: ADM for Group Sparsity

Initialize $\mathbf{Z}, \Lambda_1, \Lambda_2, \beta_1, \beta_2 > 0$, step lengths $\gamma_1, \gamma_2 > 0$

While *stopping criterion is not met* **do**

$\mathbf{C}_e \leftarrow (\beta_1 \mathbf{I} + \beta_2 \mathbf{B}^T_{e\_prior})^{-1} \times$
$\quad (\beta_1 \mathbf{Z} - \Lambda_1 + \beta_2 \mathbf{B}^T_{e\_prior}(\mathbf{Y} - \mathbf{C}_e \mathbf{C}_b) - \mathbf{B}^T_{e\_prior}\Lambda_2)$

$\mathbf{C}_b \leftarrow (2\lambda \mathbf{I} + \beta_2 \mathbf{B}^T_{b\_prior}\mathbf{B}_{b\_prior})^{-1} \times$
$\quad (2\lambda \mathbf{C}_{b\_prior} + \mathbf{B}^T_{b\_prior}\Lambda_2 + \beta_2 \mathbf{B}^T_{b\_prior}(\mathbf{Y} - \mathbf{B}_{e\_prior}\mathbf{C}_e))$

$\mathbf{Z} \leftarrow shrink(\mathbf{C}_e + \frac{\lambda_1}{\beta_2}, \frac{w}{\beta_2})$

$\Lambda_1 \leftarrow \Lambda_1 - \gamma_1 \beta_1 (\mathbf{Z} - \mathbf{C}_e)$

$\Lambda_2 \leftarrow \Lambda_2 - \gamma_2 \beta_2 (\mathbf{B}_e \mathbf{C}_e + \mathbf{B}_b \mathbf{C}_b - \mathbf{Y})$

REFERENCES

Cai, J. F., Osher, S., and Shen, Z. (2009), Split bregman methods and frame based image restoration, *Multiscale modeling and simulation*, 8, 2, 337–369

Goldstein, T. and Osher, S. (2009), The split bregman method for l1-regularized problems, *SIAM Journal on Imaging Sciences*, 2, 2, 323–343
